# Transcriptional response to sulfide in the Echiuran Worm *Urechis unicinctus* by digital gene expression analysis

**DOI:** 10.1186/s12864-015-2094-z

**Published:** 2015-10-21

**Authors:** Xiaolong Liu, Litao Zhang, Zhifeng Zhang, Xiaoyu Ma, Jianguo Liu

**Affiliations:** Ministry of Education Key Laboratory of Marine Genetics and Breeding, College of Marine Life Sciences, Ocean University of China, Qingdao, 266003 China

**Keywords:** Digital gene expression analysis, Sulfide, Transcriptional profile, *Urechis unicinctus*

## Abstract

**Background:**

*Urechis unicinctus*, an echiuran worm inhabiting the U-shaped burrows in the coastal mud flats, is an important commercial and ecological invertebrate in Northeast Asian countries, which has potential applications in the study of animal evolution, coastal sediment improvement and marine drug development. Furthermore, the worm can tolerate and utilize well-known toxicant-sulfide. However, knowledge is limited on the molecular mechanism of *U. unicinctus* responding to sulfide due to deficiency of its genetic information.

**Methods:**

In this study, we performed Illumina sequencing to obtain the first *Urechis unicinctus* transcriptome data. Sequenced reads were assembled and then annotated using blast searches against Nr, Nt, Swiss-Prot, KEGG and COG. The clean tags from four digital gene expression (DGE) libraries were mapped to the *U. unicinctus* transcriptome. DGE analysis and functional annotation were then performed to reveal its response to sulfide. The expressions of 12 candidate genes were validated using quantitative real-time PCR. The results of qRT-PCR were regressed against the DGE analysis, with a correlation coefficient and p-value reported for each of them.

**Results:**

Here we first present a draft of *U. unicinctus* transcriptome using the Illumina HiSeq^TM^ 2000 platform and 52,093 unique sequences were assembled with the average length of 738 bp and N50 of 1131 bp. About 51.6 % of the transcriptome were functionally annotated based on the databases of Nr, Nt, Swiss-Prot, KEGG and COG. Then based on the transcriptome, the digital gene expression analysis was conducted to examine the transcriptional response to sulfide during 6, 24 and 48 h exposure, and finally 1705, 1181 and 1494 tag-mapped genes were identified as differentially expressed genes in the 6-h, 24-h and 48-h libraries, then were further subjected to pathway analyses.

**Conclusions:**

In the DGE database of *U. unicinctus*, the alterations in certain known sulfide-related pathways indicate similar changes in response to sulfide. For more than 80 % of the identified pathway members, this is the first report on their association with sulfide stress, among which glycolysis pathway and PIDD involving pathways were unique and discussed in details, and were thought to play important roles in the sulfide tolerance of *U. unicinctus*. All the results are helpful to explain the mechanism of sulfide tolerance and detoxification.

**Electronic supplementary material:**

The online version of this article (doi:10.1186/s12864-015-2094-z) contains supplementary material, which is available to authorized users.

## Background

Exogenous sulfide, a common environmental toxin, harms organisms by reducing the affinity of hemoglobin for oxygen [1], inhibiting the cytochrome c and succinate oxidase complexes [2, 3], depolarizing mitochondria [4], inducing apoptosis [5], and causing oxidative damage to RNA and DNA [6]. Moreover, sulfide is produced by mammals in vivo [7, 8] and endogenous sulfide is involved in multiple physiological functions, including inflammation [9], up-regulation of the antioxidant system [10], and activation of K_ATP_ channels [11]. Thus, exploring the physiological effects of sulfide and organismal sulfide tolerance mechanisms is important.

Responses to sulfide exposure are distinct in different organisms. The nematode *Caenorhabditis elegans* survives in the presence 50 ppm sulfide, which increases its thermotolerance and lifespan [12]. Mice exposed to 80 ppm sulfide enter a hibernation-like state to overcome the conditions inducing a low metabolic rate and decreased core body temperature without any apparent ill effects [13]. Organisms living in sulfide-rich habitats adopt a variety of defensive responses to avoid prolonged toxic exposure to sulfide; for example, endosymbiotic bacteria sulfide metabolism and mitochondrial sulfide oxidation are two primary strategies for sulfide tolerance in invertebrates [14]. The hydrothermal vent tube worm *Riftia pachyptila* and the clam *Solemya velum* use hemoglobin to deliver sulfide to their endosymbiotic bacteria [15–17]. Metatranscriptome pyrosequencing of the endosymbiotic bacteria of the clam *S. velum* revealed 28 genes involved in diverse pathways associated with sulfide metabolism, including the dissimilatory sulfite reductase pathway, the adenosine-5′-phosphosulfate pathway and the sulfur oxidation pathway [18]. For some species that lack endosymbionts, such as echiuran worm *Urechis unicinctus*, mitochondrial sulfide oxidation is important for their survival in sulfide-rich habitats, and several key enzymes, including sulfide:quinone oxidoreductase (SQR), sulfur dioxygenase (SDO), and sulfur transferase (ST), play important roles in sulfide detoxification [19–22]; however, due to the lack of sufficient high-throughput molecular data, the full complement of genes and signalling pathways involved in sulfide tolerance and utilization and the mechanisms that regulate these pathways are unknown.

The echiuran worm, *U. unicinctus*, inhabiting “U-shaped” burrows in intertidal and subtidal mudflats, is widely distributed in Russia, Japan, the Korean Peninsula and China, which belongs to phylum Echiura, a special group of invertebrate protostomes. Preliminary studies have been presented that the worm *U. unicinctus* is able to metabolize exogenous sulfide and utilize it as energy [23–25]. Moreover, characterizations of the key genes for mitochondrial sulfide oxidation [21, 22, 26–28] have been revealed. Here, a high-throughput methods—digital gene expression (DGE) technique was conducted to analyze the transcriptional response to sulfide in *U. unicinctu*s. Before the DGE analysis, the transcriptome data of *U. unicinctus* is necessary, so we performed a large-scale transcriptome sequencing of *U. unicinctus* by Illumina HiSeq^TM^ 2000 platform (BGI, Shenzhen, China) to obtain the first version of the *U. unicinctus* transcriptome.

The aims of the present study are to (i) characterize the *U. unicinctus* transcriptome; (ii) identify the genes and signalling pathways responding to sulfide in *U. unicinctus*. The results will provide a comprehensive understanding for further exploring the molecular mechanism of sulfide metabolism in *U. unicinctus*.

## Results

### Transcriptome analysis

**Fig. 1:**
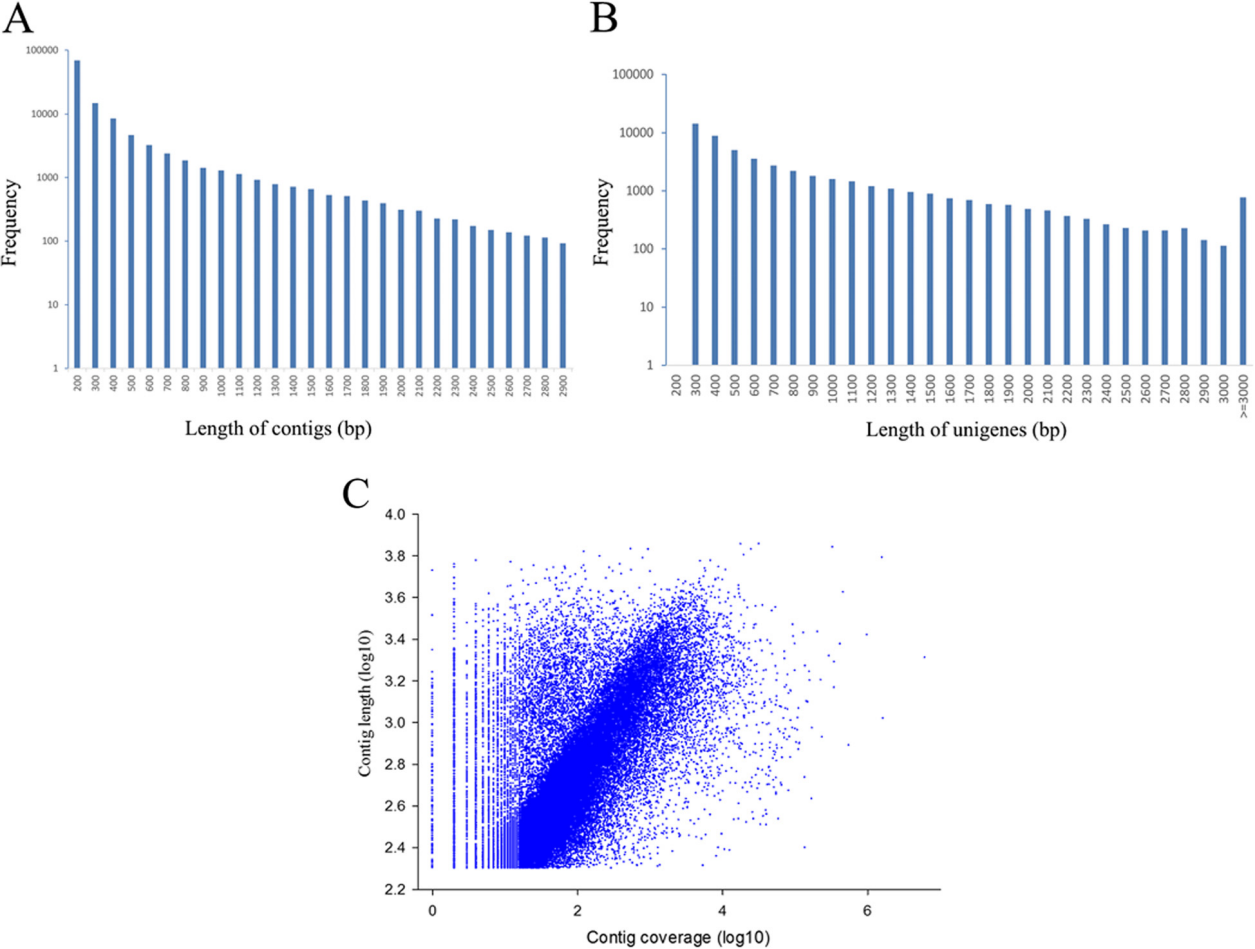
Overview of the *de novo* assembly for the *Urechis unicinctus* transcriptome. **a** Size distribution of contigs; **b** size distribution of unigenes; and **c** log-log plot showing the dependence of contig lengths on the number of reads assembled into each contig

A total of 59,007,614 raw PE reads in the fastq format were generated with a length of 2 × 90 bp, which have been deposited in the NCBI database (accession number: SRA122323). After removing the low quality reads, we obtained a total of 53,874,422 high-quality PE reads for optimizing the *de novo* assembly and analysis of the *U. unicinctus* transcriptome. The GC content, Q20 (Phred quality score >20) and unknown bases were 45.54, 97.91 and 0.00 %, respectively (Table 1). Assembly of the 53,874,422 high-quality reads generated 115,682 contigs with an average length of 337 bp, ranging from 101 to 5279 bp (Fig. 1a). Approximately 75 % (38,984,834) of the high-quality reads contributed to the assembly of the contigs. Among these contigs, 46,653 (41 %) were longer than 200 bp, and 18,663 (16 %) were longer than 500 bp. As expected for a randomly fragmented transcriptome, a positive relationship was determined between the length of a given contig and the number of reads mapped into it (Fig. 1c). Furthermore, these contigs were assembled into 52,093 unigenes with an average length of 738 bp and an N50 of 1131 bp, ranging from 200 to 7184 bp (Fig. 1b). An overview of the sequencing and assembly process is presented in Table 2.

**Table 1:** Output statistics of sequencing

Total raw reads	Total clean reads	Total clean nucleotides (nt)	Q20 percentage	N percentage	GC percentage
59,007,614	53,874,422	4,848,697,980	97.91 %	0.00 %	45.54 %

**Table 2:** Summary of the *de novo* assembly for *U. unicinctus* transcriptome

Category	Count
Total number of raw reads	59,007,614
Total number of clean reads	53,874,422
Total clean nucleotides (nt)	4,848,697,980
Average clean read length (bp)	90
Total number of contigs	115,682
Mean length of contigs (bp)	337
Contig size N50 (bp)	616
Total number of distinct clusters	12,333
Total number of distinct singletons	39,760
Total number of distinct unigenes	52,093
Unigene size N50 (bp)	1131
Sequences with E-value <10^−5^	26,200

**Fig. 2:**
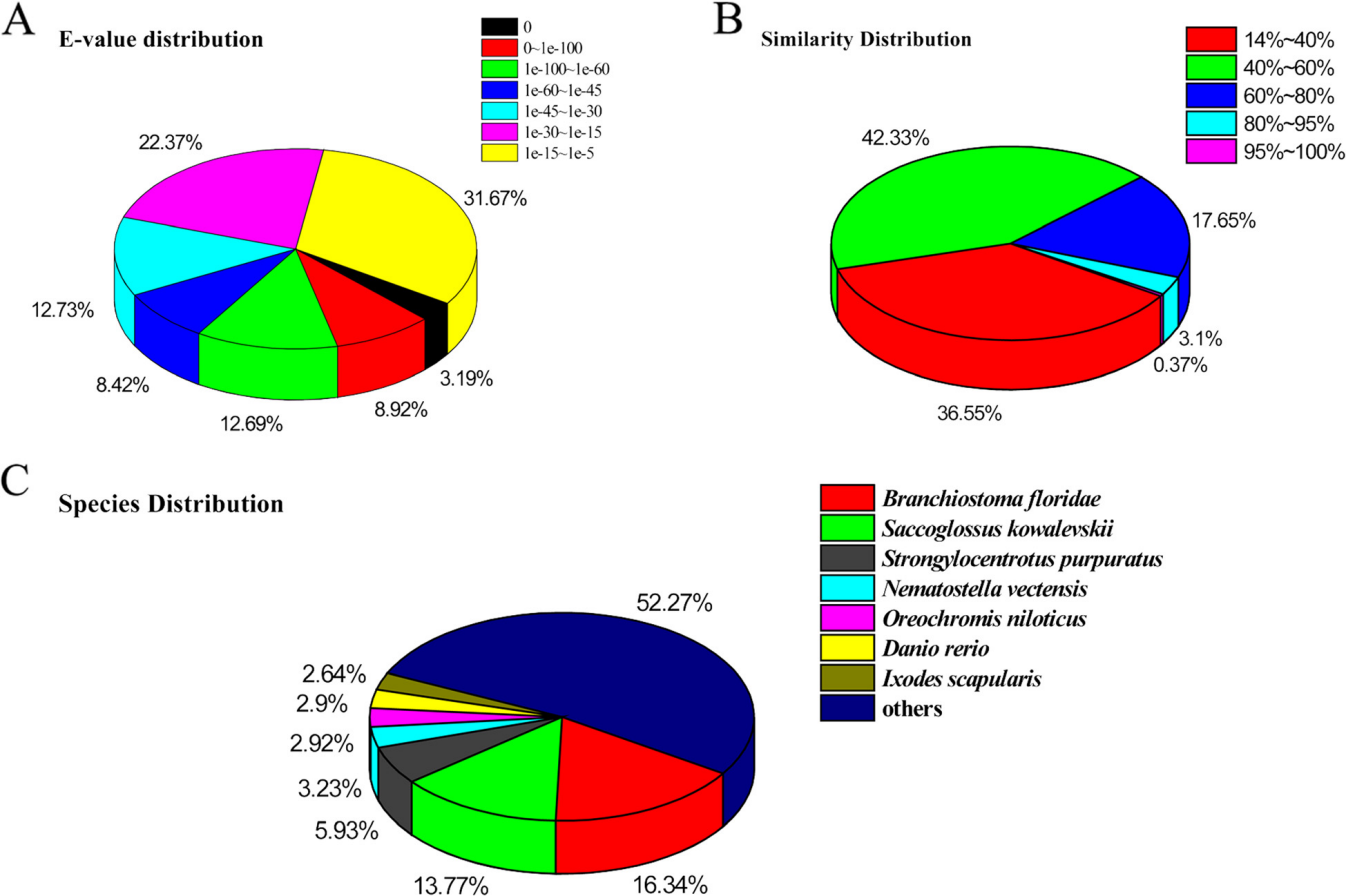
Characteristics of the homology search of illumina sequences against the Nr database. **a** E-value distribution of BLAST hits for each unique sequence with a cut-off E-value of 10^−5^. **b** Similarity distribution of the top BLAST hits for each sequence. **c** The species distribution is shown as a percentage of the total homologous sequences with an E-value of at least 10^−5^. We use the first hit of each sequence for analysis

In this study, 26,889 gene sequences (51.6 % of the total consensus sequences) returned an above cut-off BLAST result, and 51.4 % of the assembled sequences could be matched to the known proteins. There were 8707 mapped sequences (33.2 %) with high homology (*E* < 10^−45^) in the Nr database, whereas the others ranged from 10^−5^ to 10^−45^ (Fig. 2a). More than 21 % of the total sequences had a similarity that was higher than 60 %, while the rest ranged from 14 % to 60 % (Fig. 2b). Moreover, 16.34 % of the distinct sequences had top matches with those from *Branchiostoma floridae*, which was followed by *Saccoglossus kowalevskii* (13.77 %) and *Strongylocentrotus purpuratus* (5.93 %) (Fig. 2c).

**Fig. 3:**
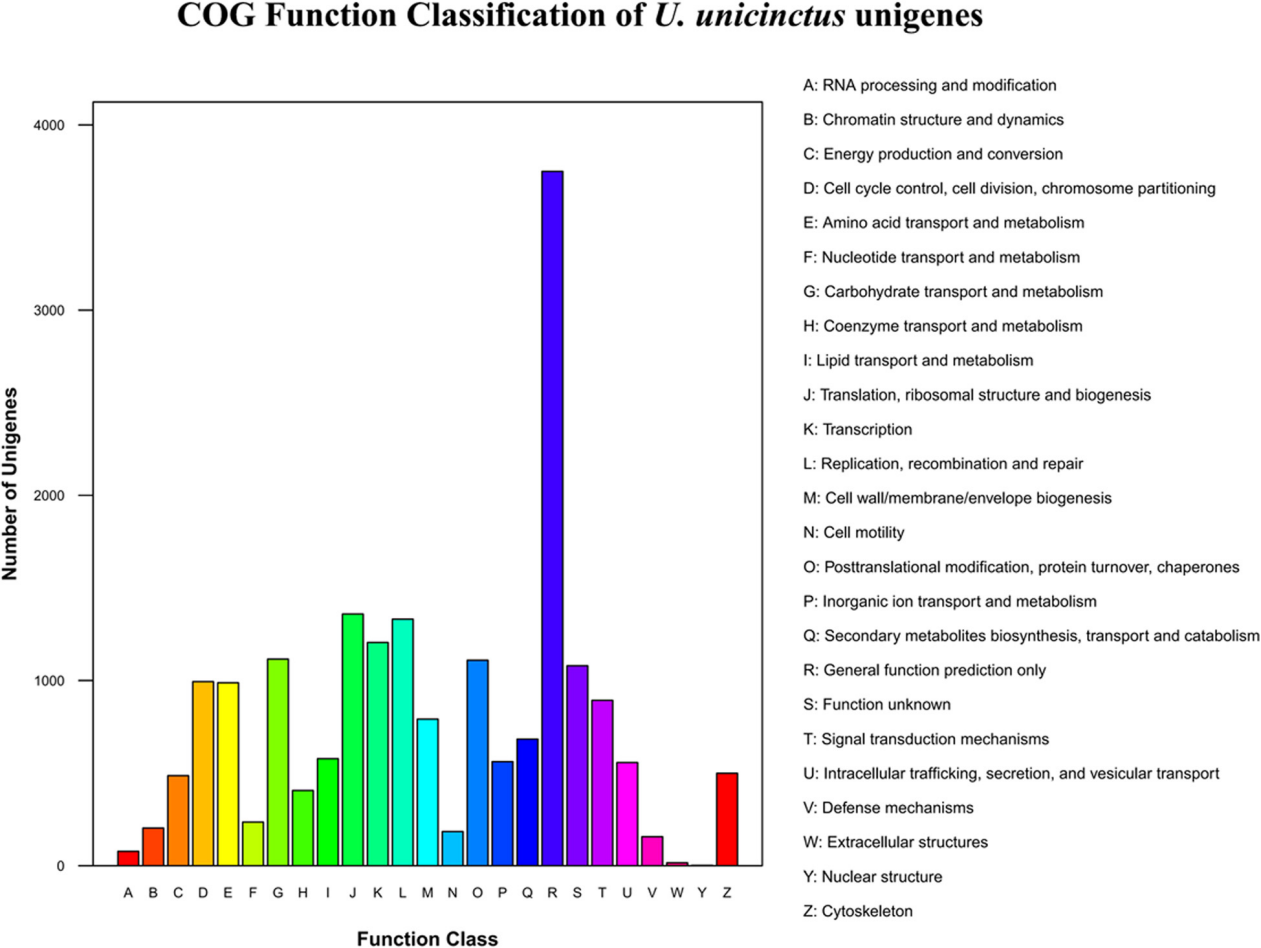
Histogram presentation of clusters of orthologous group (COG) classification. Out of 26,889 annotated sequences, 9447 sequences have a COG classification among the 25 categories

COG (Cluster of orthologous Groups of proteins) analysis showed that 9447 unigenes were classified into 25 categories assigned by COG (Fig. 3). In this classification, the largest group was involved in the general function, containing 3749 unigenes (39.68 %), which was followed by translation, ribosomal structure and biogenesis (1360, 14.40 %); replication, recombination and repair (1332, 14.10 %); transcription (1206, 12.77 %), carbohydrate transport and metabolism (1116, 11.81 %); and posttranslational modification, protein turnover, and chaperones (1110, 11.75 %). Only 2 unigenes (0.02 %) belong to the nuclear structure, which was the smallest group.

**Fig. 4:**
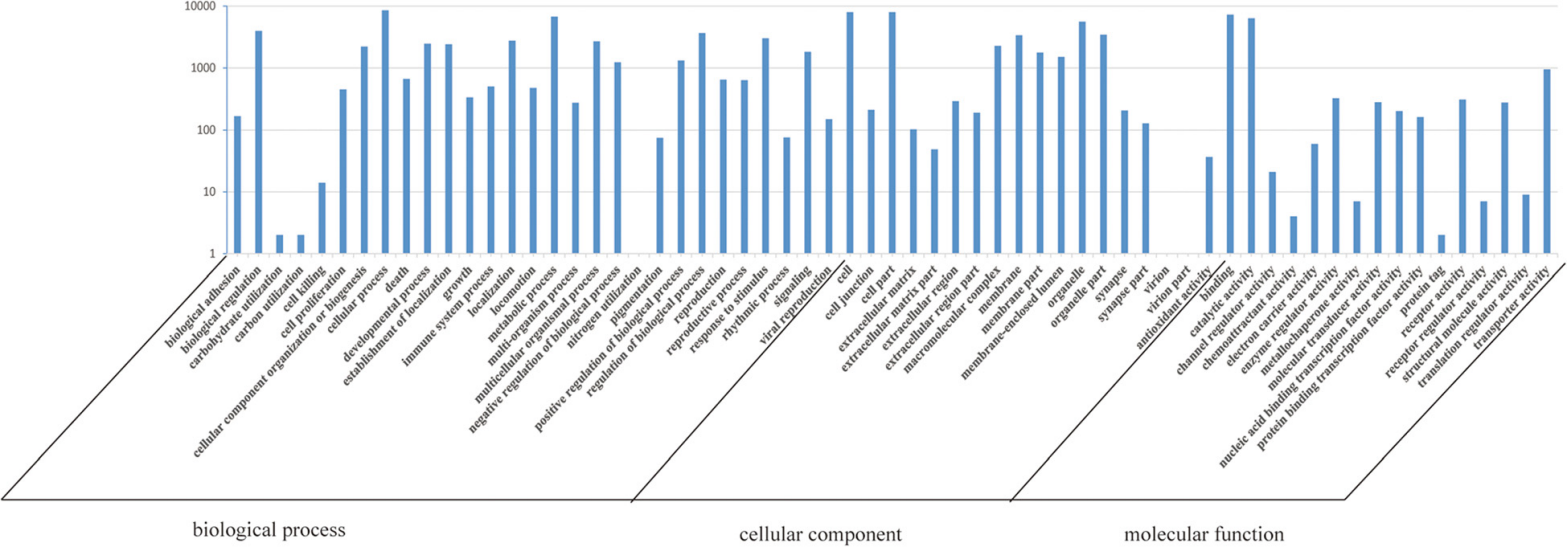
Histogram presentation of the Gene Ontology classification. The results are summarized in the following three main categories: biological process, cellular component and molecular function. The right y-axis indicates the number of genes in a category. The left y-axis indicates the percentage of a specific category of genes in that main category

GO (Gene ontology) analysis showed that a total of 13,458 unigenes (51.37 % of the total annotated sequences) were assigned at least one GO term. The transcripts were divided into 63 functional groups within the following three ontologies: molecular function, cellular component and biological process. For the biological process, the most representations were for cellular process (8576, 63.72 % of the total 13,458) and metabolic process (6796, 50.50 %); the cell (7985, 59.33 %), cell part (7979, 59.29 %) and organelle (5601, 41.62 %) for the cellular component; and the binding (7276, 50.04 %) and catalytic activity (6347, 47.16 %) for the molecular function, respectively. Only 60 and 9 unigenes were predicted to act in the functional group electron carrier and translation regulator activity, which were the smallest two parts (Fig. 4).

KEGG (Kyoto Encyclopedia of Genes and Genomes) pathway analysis showed that 18,884 unigenes (72.73 % of the total annotated sequences) were mapped to 254 KEGG pathways. Of these, metabolic pathways contained 2820 unigenes and were no doubt larger than other pathways, such as pathways in focal adhesion (717), cancer (654) and regulation of the actin cytoskeleton (645). Approximately 18 % of the unigenes were related to such pathways as immunity, stress, and growth.

### DGE library sequencing and tag mapping

To investigate the transcriptome profile of *U. unicinctus* in response to sulfide at 6, 24 and 48 h, DGE analysis was performed. There were a total of 3.8–4.2 million raw tags. After removing the adaptor tags and the low-quality tags, more than 95 % of the raw tags were retained, including 3,909,160, 3,613,737, 4,057,279 and 4,027,932 clean tags for the control, 6-h, 24-h and 48-h libraries, respectively (Table 3). These results suggested that the sequencing data were of sufficient quality for subsequent analysis.

**Table 3:** DGE sequencing statistics

Summary		Control 0 h	Exposure to Sulfide		
			6 h	24 h	48 h
Raw data	Total number	4,089,259	3,772,420	4,214,487	4,218,738
	Distinct tag number	223,037	194,769	183,978	196,142
Clean tags	Total number	3,909,160	3,613,737	4,057,279	4,027,932
	Distinct tag number	94,189	86,979	82,866	82,991
All tags mapping to a gene	Total number	3,381,053	2,982,514	3,406,833	3,372,408
	Total % of clean tags	86.49 %	82.53 %	83.97 %	83.73 %
	Distinct tag number	60,344	49,296	49,136	47,831
	Distinct tag % of clean tags	64.07 %	56.68 %	59.30 %	57.63 %
Unambiguous tags mapping to a gene	Total number	2,843,143	2,484,170	2,865,778	2,839,135
	Total % of clean tag	72.73 %	68.74 %	70.63 %	70.49 %
	Distinct tag number	48,405	39,557	39,685	38,365
	Distinct tag % of clean tags	51.39 %	45.48 %	47.89 %	46.23 %
All tag-mapped genes	Number	21,121	20,526	20,129	20,111
	% of reference genes	40.54 %	39.40 %	38.64 %	38.61 %
Unambiguous tag-mapped genes	Number	14,722	14,264	13,937	13,832
	% of reference genes	28.26 %	27.38 %	26.75 %	26.55 %
Unknown tags	Total number	502,708	591,656	601,170	601,065
	Total % of clean tags	12.86 %	16.37 %	14.82 %	14.92 %
	Distinct tag number	32,399	35,742	31,937	33,194
	Distinct tag % of clean tags	34.40 %	41.09 %	38.54 %	40.00 %

The raw data are tags obtained from sequencing without processing. The clean tags are tags that remained after filtering out dirty (low-quality) tags from the raw data. The distinct tags are tags that do not overlap. The unambiguous tags are the clean tags that remained after the removal of tags mapped to reference sequences from multiple genes

**Fig. 5:**
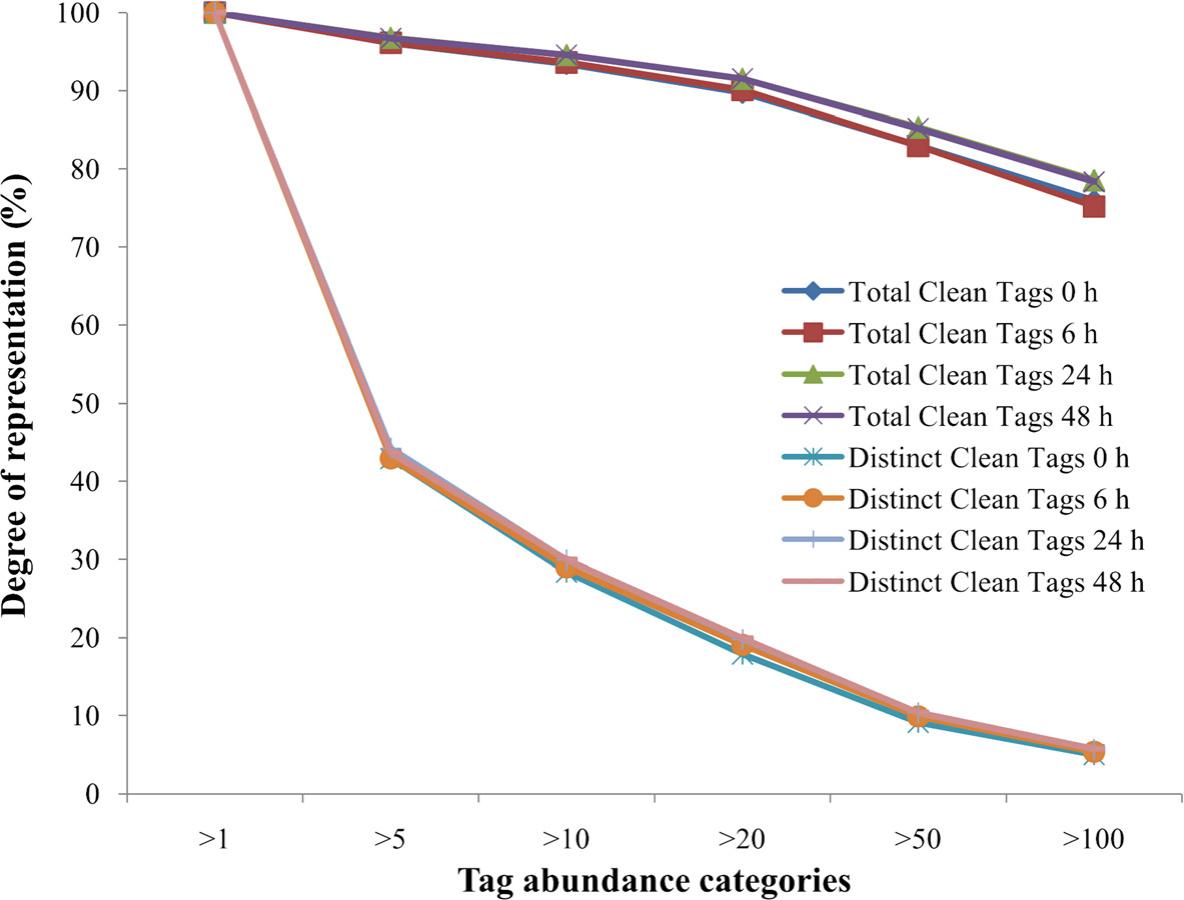
Distribution of the total clean tags and distinct clean tags in *Urechis unicinctus* exposed to 50 μM sulfide for different periods

**Fig. 6:**
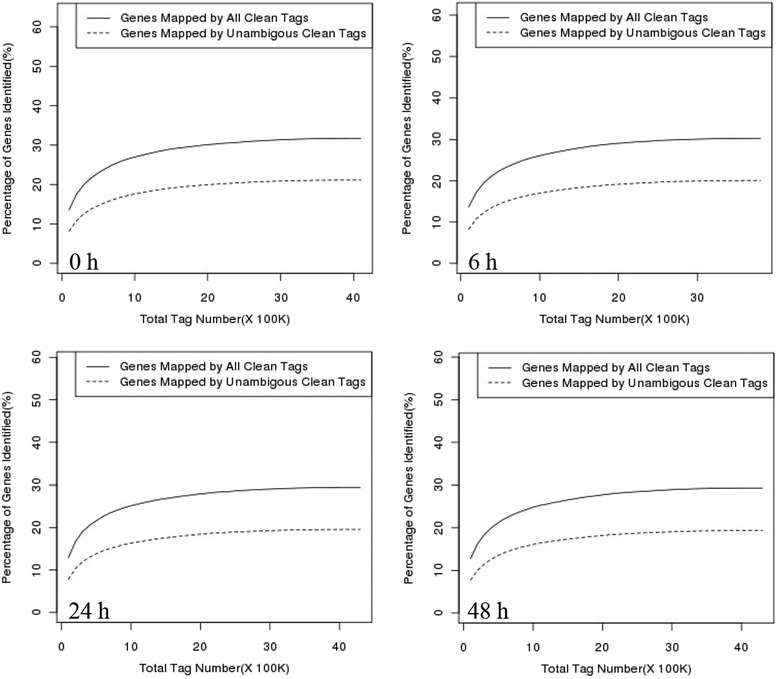
Sequencing saturation analysis of *Urechis unicinctus* exposed to 50 μM sulfide for different periods

The distribution of the total clean tags and the distinct clean tags in each tag-abundance category was similar among the four DGE libraries (Fig. 5). Sequencing saturation analysis of the four libraries revealed that the number of detected genes plateaued shortly after 1 M tags were sequenced, and no new genes were identified when the total sequence number approached 2 M, indicating that the approximately 4 M tags in each library represented complete coverage of the transcriptome (Fig. 6).

To elucidate the molecular events underlying the DGE profiles, the clean tags were mapped to the previous transcriptome. In the control, 6-h, 24-h and 48-h libraries, 51.39, 45.48, 47.89 and 46.23 % of the distinct clean tags uniquely mapped to the reference sequence, generating 21,121, 20,526, 20,129 and 20,111 tag-mapped genes, respectively (Table 3).

### DEG (differentially expressed gene) identification, validation and pathway enrich analysis

**Fig. 7:**
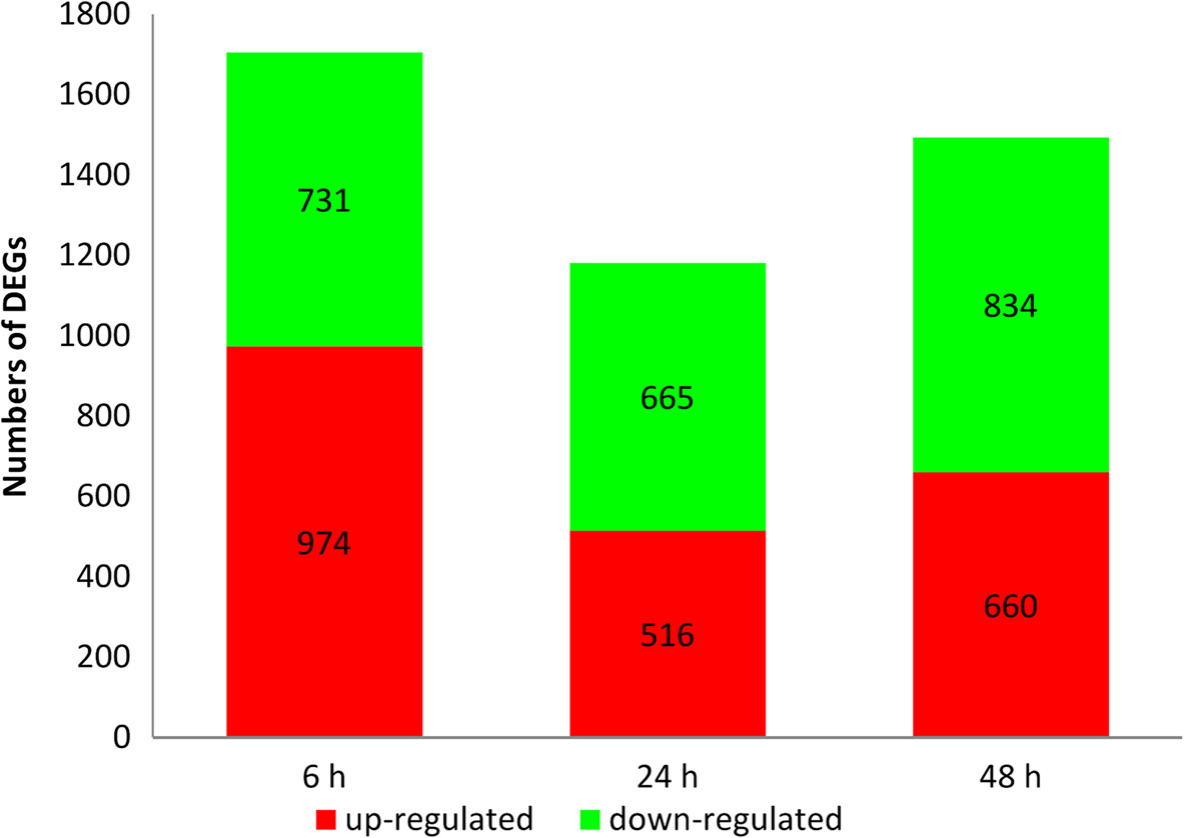
DEG transcriptome profiles of *Urechis unicinctus* exposed to 50 μM sulfide for different periods. The values in the *red* and *green* regions represent the number of up-regulated and down-regulated DEGs, respectively

Compared to the control library, 1705, 1181 and 1494 tag-mapped genes were identified as DEGs (false discovery rate (FDR) ≤ 0.001 and fold-change cut-off of 2 fold) in the 6-h, 24-h and 48-h libraries, respectively (Additional file 1). In the 6-h library, 974 genes (57.1 % of the 6-h library DEGs) were up-regulated, and 731 (42.9 % of the 6-h library DEGs) were down-regulated. A majority of the DGEs in the 24-h and 48-h libraries were down-regulated (665 (56.3 %) of the 24-h library DEGs and 834 (55.8 %) of the 48-h library DEGs) (Fig. 7).

**Fig. 8:**
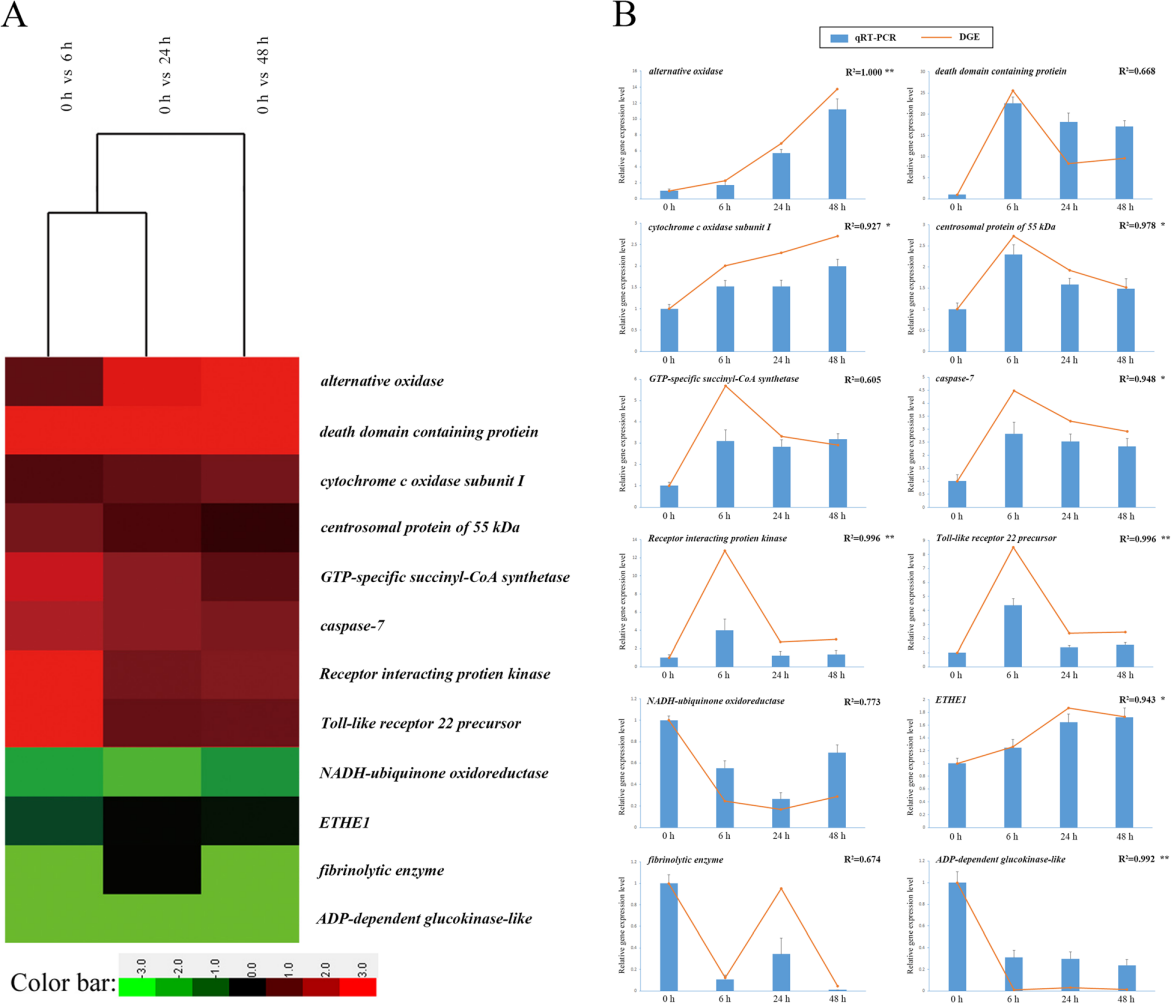
qRT-PCR verification of the digital gene expression profiles of selected DEGs. **a** DGE; **b** qRT-PCR results versus DGE results. For each qRT-PCR validation, five technical replications were performed. The correlation coefficient (R^2^) of the regression between qRT-PCR and DGE for each of the 12 unigenes investigated was analyzed and shown. *p < 0.05 and **p < 0.01

To validate the digital gene expression (DGE) results, 12 candidate unigenes representing different biological processes were selected for qRT-PCR analysis. The results of qRT-PCR were regressed against the DGE analysis, with a correlation coefficient (R^2^) and *p*-value reported for each of the 12 unigenes investigated (Fig. 8). Of the selected unigenes, eight returned a correlation coefficient with a *p*-value < 0.05 or <0.01, showing the consistence between DGE and qRT-PCR. The left 4 unigenes did not show a significant correlation. However, all the candidate unigenes demonstrated a concordant trend of change for both DGE and qRT-PCR results, indicating that our results were reliable.

KEGG pathway analysis of the DEGs revealed that 175, 166 and 176 metabolic pathways were affected in *U. unicinctus* exposed to 50 μM sulfide for 6, 24, and 48 h, respectively and the pathways including oxidative phosphorylation, ribosome, proteasome, complement and coagulation cascades, and metabolism were all significantly enriched in the 6-h, 24-h and 48-h library (Additional file 2).

## Discussion

*U. unicinctus*, belonging to the phylum Echiura, can tolerate high concentrations of sulfide living in the “U-shaped” burrows in intertidal and subtidal mudflats. In this study, we first demonstrated a *de novo* assembly of the *U. unicinctus* transcriptome using Illumina Hiseq2000 platform. This is also the first transcriptome report in echiuran worms. In the obtained transcriptome of *U. unicinctus*, the assembly of the 53,874,422 clean reads produced 52,093 unique sequences with an average size of 738 bp which is comparable with other studies generating transcriptome data [29–31]. The number of protein-coding genes in the *U. unicinctus* genome is unknown; however, if we assume general conservation based on estimates for the representative of the phylum Annelida *Capitella teleta* (32,415 genes; JGI annotation pipeline), ~83 % of protein-coding genes (26,889) were assembled in this study. All the transcriptome data obtained in *U. unicinctus* indicated that it is appropriate to conduct DGE analysis to identify transcriptional response to sulfide in *U. unicinctus*.

In the DGE analysis, a total of 1705, 1181 and 1494 tag-mapped DEGs were identified, and 175, 166 and 176 metabolic pathways were affected when the worms were exposed to 50 μM sulfide for 6, 24 or 48 h, respectively. These DEGs displayed many biological functions, suggesting molecular mechanisms involved in the sulfide response. In mammals, several pathways—such as MAPK, NF-κB and apoptosis—are affected by sulfide and might be important for the sulfide tolerance [5, 9, 32–37]; we also identified some DEGs in these pathways such as ribosomal protein S6 kinase 2 alpha (RSK2) and phospholipase A2 (cPLA2) in the MAPK pathway, Toll-like receptor 4 (TLR4) and myeloid differentiation primary response protein 88 (Myd88) in the NF-κB pathway and caspase-3 and caspase-7 in the apoptosis pathway (Table 4), which are consistent with the previous results. Furthermore, in fission yeast *Schizosaccharomyces pombe*, it is revealed by microarray analysis that a large number of genes encoding mitochondrial proteins were down regulated after sulfide treatment, such as NADH dehydrogenase, succinate dehydrogenase, sulfide-quinone oxidoreductase (*sqr*), glycerol-3-phosphate dehydrogenase (*gut*), ubiquinol cytochrome c reductase complex subunit 5 (*rip1*) and cytochrome c oxidase subunit VIA (*cox13*), indicating that sulfide could affect the expressions of many mitochondrial proteins at transcriptional level [38]. In our DGE results, the mRNA expression levels of genes encoding mitochondrial complex I (NADH dehydrogenase) and complex II (succinate dehydrogenase) were down-regulated (Table 4), which was similar with the results of [38]. Meanwhile the expression of genes such as *sqr*, *sdo* and *st* encoding mitochondrial sulfide oxidation enzymes increased with the sulfide exposure time (Table 4), which agreed with our previous studies [26, 27]. These results demonstrate that our databases are reliable.

**Table 4:** DEGs and their associated pathways in *U. unicinctus* exposed to sulfide

Pathway	*U. unicinctus* Unigene accession number	Gene annotation	Differential expression multiplication log_2_ Ratio (TPM time/ TPM control)		
			6 h	24 h	48 h
Apoptosis					
	Unigene6112_No.2	caspase-3	2.23	2.90	1.85
	CL530.Contig1_No.2	caspase-7	2.16	1.73	1.54
Glycolysis					
	CL3519. Contig1_No.2	pyruvate kinase	−0.64	−1.08	−0.85
	Unigene2735_No.2	phosphorylase	−0.43	−1.08	−0.58
	CL1308. Contig1_No.2	phosphofructokinase	−0.70	−0.59	−1.08
	Unigene1913_No.2	ADP-dependent glucokinase	−6.77	−4.93	−6.34
	CL5108.Contig1_No.2	phosphoglycerate kinase	−1.68	−1.61	−1.40
MAPK					
	Unigene2294_No.2	cPLA2	3.38	4.17	2.67
	Unigene1096_No.2	RSK2	3.61	2.12	2.40
NF-κB					
	Unigene8158_No.2	TLR4	3.09	1.24	1.30
	Unigene15896_No.2	Myd88	3.06	1.95	2.27
	Unigene3552_No.2	NEMO	1.45	0.94	0.61
	Unigene5808_No.2	RIP1	3.68	1.44	1.59
Oxidative phosphorylation					
	Unigene1548_No.2	NADH dehydrogenase [ubiquinone] 1 alpha subcomplex subunit 2-like	−1.76	−1.07	−1.48
	Unigene1556_No.2	NADH-ubiquinone oxidoreductase	−2.01	−2.56	−1.79
	Unigene2512_No.2	NADH dehydrogenase	−8.48	−8.48	−8.48
	Unigene1586_No.2	NADH dehydrogenase [ubiquinone] 1 beta subcomplex subunit 9-like	−1.13	−0.75	−0.87
	Unigene2306_No.2	succinate dehydrogenase [ubiquinone] iron-sulfur subunit	−1.24	−0.33	−0.31
	Unigene1680_No.2	ubiquinol-cytochrome c reductase	1.06	0.68	0.44
	Unigene1299_No.2	cytochrome c oxidase subunit I	1.00	1.21	1.43
	Unigene1383_No.2	mitochondrial H+ ATPase a subunit	1.19	0.51	0.47
p53					
	CL5080.Contig1_No.2	PIDD	4.68	3.07	3.26
Sulfide oxidation					
	Unigene2733_No.2	sulfide-quinine oxidoreductase	0.35	0.47	1.30
	Unigene4395_No.2	sulfur dioxygenase	−0.83	−0.10	−0.20
	Unigene1081_No.2	sulfur transferase	0.13	0.27	0.20

In this study, several unique pathways and more than 80 % of the identified pathway members are the first report, which are associated with sulfide stress in *U. unicinctus* exposed to sulfide. Among these pathways, ATP generated pathways (glycolytic and oxidative phosphorylation pathway) as well as the PIDD (p53-induced protein with a death domain) induced pathways involving in the DNA damage response attracted our interests.

Usually the anaerobic glycolytic pathway typically replaces oxidative phosphorylation for ATP production and the maintenance of the intracellular ATP balance when mouse or human cells are cultured in media containing 10 μM sulfide, and the expression of nearly all of the genes in the anaerobic glycolytic pathway are up-regulated [39, 40]. In *U. unicinctus*, it is confirmed that activities of key enzymes in the glycolytic pathway, phosphorylase, phosphofructokinase, and pyruvate kinase become markedly high after fertilization in *U. unicinctus* eggs [41, 42], and enzymes associated with oxidative phosphorylation for ATP production have also been elucidated in echiuran respiration [43–47]. In this study, we identified a large number of genes related to glycolytic and oxidative phosphorylation pathways, such as pyruvate kinase, phosphorylase, phosphofructokinase, ADP-dependent glucokinase and phosphoglycerate kinase (Table 4). Interestingly, the expressions of most unigenes involved in glycolysis such as ADP-dependent glucokinase and phosphoglycerate kinase were significantly down-regulated when the worms were exposed to sulfide (Table 4); moreover, the mRNA levels of both complex I and complex II were also down-regulated (Table 4). These results suggested unlike mammals, *U. unicinctus* may adopt a non-glycolytic pathway to produce ATP when sulfide exposure. Several sulfide-tolerant organisms—such as the ribbed mussel *Geukensia demissa*, echiuran worm *U. unicinctus* and the lugworm *Arenicola marina*—utilize sulfide to produce ATP [24, 48–50]. Hildebrandt [51] suggested that a super-complex composed of SQR and complexes III and IV initiates ATP production using sulfide as a material resource when sulfide enters the respiratory chain of rat mitochondria. In this study, the genes encoding SQR and complexes III and IV (Table 4) were all up-regulated in *U. unicinctus* exposed to sulfide. Therefore, we propose that the super-complex (SQR, complexes III and IV) catalytic activity by using sulfide as a material resource replaces the glycolysis to maintain the ATP balance when these worms are exposed to sulfide. Further studies are needed to confirm these suggestions.

It is known sulfide generates significant oxidative damage to genomic DNA [52]. Typically, PIDD, a key member of the p53 DNA damage response pathway, activates two distinct pathways in response to DNA damage. One is to activate NF-κB via the ubiquitination of NEMO to stimulate DNA repair and promote organismal survival [53–55]; alternatively, PIDD induces genotoxic stress-mediated apoptosis and activates the mitochondrial apoptosis pathway to protect genome integrity [56]. In this study, all the members involving in this pathway were all in up-regulated expression when *U. unicinctus* were exposed to sulfide (Table 4). Furthermore, it is suggested that PIDD acts in response to sulfide-induced DNA damage based on probably the increase of the *PIDD* mRNA level. Further researches need to be conducted to verify the suggestions from the DGE analysis.

## Conclusions

In this study, a transcriptome from the *U. unicinctus* adult tissues was sequenced. This database is currently the largest in echiuran worms, and 52,093 unigenes are assembled. Many unigenes have been identified and are involved in various fields. Then based on the transcriptome, transcriptional profile of *U. unicinctus* exposed to 50 μM was revealed via DGE analysis. Approximately 1705, 1181 and 1494 DEGs were identified at 6, 24 and 48 h after sulfide exposure, respectively. These DEGs are involved in many biological processes. In the DGE database of *U. unicinctus*, the alterations in certain known sulfide-related pathways indicate similar changes in response to sulfide, whereas *U. unicinctus* exhibited differences from other species with respect to its modulation of glycolysis in response to sulfide. In addition, this is the first report on *PIDD* in response to sulfide-induced DNA damage in *U. unicinctus*. These data facilitates the identification of sulfide-related genes and elucidates the molecular mechanisms by which organisms tolerate sulfide stress. This study provides a framework for additional functional studies to examine sulfide toxicology in this system.

## Methods

### Ethics statement

Each of the procedures that were used to handle and treat the Echiuran worms during this study was approved by the Ocean University of China Institutional Animal Care and Use Committee (OUC-IACUC) prior to the initiation of the study.

### Animals and sulfide treatment

*Urechis unicinctus* (mean fresh mass of 33.4 ± 10.4 g) were purchased from an aquatic product market, which collected worms from a coastal intertidal flat in Yantai, China. The worms were temporarily maintained in aerated seawater (17 ± 1 °C, pH 8.0, and salinity 32 PSU) for a week without feeding. Then 30 healthy worms were randomly assigned to three groups (10 worms per group), and for each group the worms were maintained in a sealed aquarium containing 30 L of seawater. The sulfide concentration was maintained at 50 μM by adding a sulfide stock solution (10 mM Na_2_S, pH 8.0) every 2 h as necessary based on the sulfide concentration, which was determined using the methylene blue method [57]. Six worms were sampled (i.e., two individuals from each aquarium) before sulfide addition (control) and at 6, 24 and 48 h after the initiation of sulfide exposure, frozen in liquid nitrogen, and stored at −80 °C for subsequent RNA extraction.

### RNA extraction and quality control

Total RNAs from each stored tissues were extracted with TRIzol® Reagent (Invitrogen, Carlsbad, CA, USA) following the manufacturer’s instructions. The yield, purity and integrity of the RNA samples were analyzed using a 2100 Bioanalyzer (Agilent Technologies, Santa Clara, CA, USA).

### cDNA library construction and illumina sequencing

The mRNA was purified using a PolyATract® mRNA Isolation System (Ambion, Austin, TX, USA). Fragmentation buffer was added to interrupt mRNA to short fragments. With these short fragments as templates, random hexamer-primer was used to synthesize the first-strand cDNA. The second-strand cDNA was synthesized using buffer, dNTPs, RNase H and DNA polymerase I. Short fragments were purified with QiaQuick PCR extraction kit (Qiagen, Hilden, Germany) and resolved with EB buffer for end reparation and tailing A. Next, the short fragments were connected with sequencing adapters. After agarose gel electrophoresis, suitable fragments were selected for the PCR amplification as templates. Finally, the library could be sequenced using Illumina HiSeq™ 2000, which could generate paired-end (PE) reads with a length of 90 bp.

### De novo assembly and transcriptome annotation

Raw reads from Illumina sequencing were first pre-processed for quality control, such as filtering of high-quality reads based on the score value given in fastq files, removal of reads containing primer/adaptor sequences and trimming the read length using common programs. After comparison, the SOAP-denovo program was most effective for the assembly of 25-bp segments that are randomly clipped from the reads obtained in long parts with the help of the de Bruijn graph algorithm and was then used for de novo assembly of our PE reads and generation of non-redundant unique sequences.

Unigenes were firstly aligned for homologues by blastX (E-value < 10^−5^) to protein databases Nr, Swiss-Prot, KEGG and COG and then aligned by blastn to nucleotide databases Nt (E-value < 10^−5^). GO terms were assigned utilizing Blast2GO [58] for blast output obtained from the Nr database. After getting GO annotation for every unigene, WEGO software [59] was used to do GO functional classification for all unigenes and to understand the distribution of gene functions of the species from the macro level. With the help of KEGG database, pathway annotation for unigenes was obtained.

### DGE library construction, sequencing and annotation

Four DGE libraries were constructed from the total RNA sample of the worms exposed to 50 μM sulfide for 0, 6, 24 or 48 h. For all libraries, mRNA was purified from 6 μg of total RNA sample using oligo(dT) magnetic beads and was reverse-transcribed into double-stranded cDNA (SuperScript II, Invitrogen). Two restriction enzymes were used to generate sequencing tags. The bead-bound cDNA was digested using NlaIII (CATG recognition site) and ligated to Illumina adaptor 1. The junction of Illumina adapter 1 and CATG generated the recognition site for MmeI. Thereafter, MmeI was used to cut the cDNA fragments 17 bp downstream of the CATG site. After removing the 3′ fragment via magnetic bead precipitation, Illumina adapter 2 was introduced at the site of MmeI cleavage, resulting in a library of tagged cDNA containing different adaptors on each end. Fifteen cycles of PCR amplification were performed to enrich the 21-bp tags in the samples. Massively parallel sequencing-by-synthesis was performed using an Illumina HiSeq™ 2000. Clean tags were obtained by filtering the adaptor sequences and low-quality tags using the Illumina data processing pipeline. The distinct clean tags were classified according to their copy number in the libraries, and the saturation of the four libraries was analyzed. The distinct clean tags were mapped to the transcriptome of *U. unicinctus* sequenced before. Only the tags displaying zero or one mismatch were annotated based on the reference genes.

### Identification and validation of DEGs

To compare the gene expression in *U. unicinctus* at different periods of sulfide exposure, the number of raw clean tags in each library was first normalized to the number of transcripts per million clean tags (TPM). The expression analysis was then carried out using TPM of sulfide treatment groups compared with that of the control group. The thresholds for determining significant differences in gene expression were a FDR (false discovery rate) ≤ 0.001 and an absolute value of log_2_ Ratio ≥1. All DEGs were subjected to KEGG pathway enrichment analysis compared with the transcriptome background using hypergeometric test. The calculated P-value goes through as a threshold. KEGG pathway fulfilling corrected *P*-value ≤ 0.05 from Bonferroni correction were defined as significantly enriched terms in DEGs.

The expression of tag-mapped genes in the four libraries was validated using quantitative real-time reverse-transcription polymerase chain reaction (qRT-PCR). Primers were designed using Primer 5.0 software for 12 selected genes, including 8 up-regulated genes, 3 down-regulated genes and 1 gene not displaying differential expression (Additional file 3: Table S1). Total RNA sample (1 μg) from the stock samples used to construct the sequencing libraries was reverse-transcribed into cDNA using the Primescript™ RT Reagent Kit with gDNA Eraser (Takara, Otsu, Japan). All amplification reactions were performed using a LightCycler™ 480 (Roche, Basel, Switzerland) in a 20-μL volume containing 1 × SYBR Premix Ex Tap (Takara), 0.2 mM of each primer and 1.5 μL of cDNA. The amplification was conducted according to the following standard protocol: 95 °C for 10 min, followed by 40 cycles of 95 °C for 15 s, 60 °C for 1 min, and a thermal denaturing step to generate the melting curves for the verification of amplification specificity. The *U. unicinctus β-actin* (GenBank accession number: GU592178.1) was used as an internal standard, and the relative gene expression levels were calculated using the 2^-ΔΔCt^ method, where -ΔCt is the difference in the Ct between *β-actin* and the target gene.

## Availability of supporting data

Raw data was submitted to NCBI’s short read archive (SRA, SRA122323). The gene expression data induced by the sulfide are available in the online Supporting Information.

## Additional files

Additional file 1:
**The list of differentially expressed genes for** ***Urechis unicinctus*** **in response to sulfide.** (XLSX 606 kb) Additional file 2:
**The data of pathways analysis for differentially expressed genes of** ***Urechis unicinctus*** **in response to sulphide.** (XLSX 44 kb) Additional file 3: Table S1. qRT-PCR primers for the DEG validation. (DOCX 16 kb)
