# Supplementary material for: Transcriptional response to sulfide in the Echiuran Worm Urechis unicinctus by digital gene expression analysis
Source: BMC Genomics. 2015 Oct 21;16:829. doi: 10.1186/s12864-015-2094-z (PMC4618349; doi:10.1186/s12864-015-2094-z)
Supplement: Additional file 3: Table S1. — qRT-PCR primers for the DEG validation. (DOCX 16 kb) [file 12864_2015_2094_MOESM3_ESM.docx]

Table S1 qRT-PCR primers

| **Gene** | **Primer (5’- 3’)** | **Product (bp)** |
| --- | --- | --- |
| **CL530.Contig1_No.2** | **F**-GATTTGCTGGGTTCAGGTGC | **190** |
|  | **R**-CTAAGGACGAGAAGATACGGG |  |
| **CL5080.Contig1_No.2** | **F**-GGCGAACTATGGCAGTCTTAC | **88** |
|  | **R**-TGGCGGCTATCTCGTGAAC |  |
| **Unigene1299_No.2** | **F**-TGGAGGAGTAGGAACAGGGTG | **177** |
|  | **R**-GGTCGTAGGCCATCTCATCGT |  |
| **Unigene1556_No.2** | **F**-ACTCTTCTCCTGGCTTCGG | **193** |
|  | **R**-GGTCTGTTAGTGTTTGTGGCTC |  |
| **Unigene1913 _No.2** | **F**-GTTAGTTCCCCGCAGCTTC | **158** |
|  | **R**-CTTCAAATGCTGTCCTGGGTG |  |
| **Unigene2908_No.2** | **F**-GTGATCCCCGAGAAGTGAAG | **166** |
|  | **R**-ACAGCACCTCCAACATCCAG |  |
| **Unigene3552_No.2** | **F**-TCGGTGGATTCCTGTCTGTC | **105** |
|  | **R**-CCTCTTCAAGCATTGGATTTCG |  |
| **Unigene4161_No.2** | **F**-AGGGTTCTTCGCTTTCGGTG | **100** |
|  | **R**-GGAGGCAATCGGTAGTAGTC |  |
| **Unigene4395_No.2** | **F**-ACAGGGATGTTCGTATCGTCAA | **153** |
|  | **R**-ATTCGGCAATCACACTCTTACG |  |
| **Unigene5808_No.2** | **F**-AGCCAAACAGCCCAGCAAAC | **151** |
|  | **R**-GTTGAATGGCGGAGCGTTAC |  |
| **Unigene8158_No.2** | **F**-CCCACAGAACAACTCACCC | **121** |
|  | **R**-TACGAATGGCAGCACGAAGTC |  |
| **Unigene18034_No.2** | **F**-GTAGCCTTGCCTCATCCCATC | **165** |
|  | **R**-TTCATCTTGTCTCCCTCTGCTG |  |
